# Methyl (2*Z*)-2-bromo­methyl-3-(2,4-dichloro­phen­yl)prop-2-enoate

**DOI:** 10.1107/S1600536813007368

**Published:** 2013-03-23

**Authors:** K. Swaminathan, K. Sethusankar, Anthonisamy Devaraj, Manickam Bakthadoss

**Affiliations:** a Department of Physics, RKM Vivekananda College (Autonomous), Chennai 600 004, India; b Department of Organic Chemistry, University of Madras, Maraimalai Campus, Chennai 600 025, India

## Abstract

In the title compound C_11_H_9_BrCl_2_O_2_, which represents the *Z* isomer, the methyl­acrylate moiety is essentially planar within 0.039 (2) Å and has an extended *trans* configuration. The benzene ring makes a dihedral angle of 28.3 (1)° with the mean plane of the methyl­acrylate moiety. The crystal packing is characterized by C—H⋯O hydrogen bonding and halogen–halogen inter­actions [Cl⋯Cl = 3.486 (3) Å], resulting in the formation of *R*_2_^2^(11) ring motifs and connecting the mol­ecules into chains propagating along the *b* axis.

## Related literature

For the uses of cinnamic acid and its derivatives, see: Xiao *et al.* (2008 ▶); De Fraine & Martin (1991 ▶). For the extended conformation of acrylate, see: Schweizer & Dunitz (1982 ▶). For a related structure, see: Karthikeyan *et al.* (2012 ▶). For graph-set notation, see: Bernstein *et al.* (1995 ▶). For type I halogen inter­actions, see: Johnson & Lemmerer (2012 ▶); Schmidt *et al.* (2011 ▶).
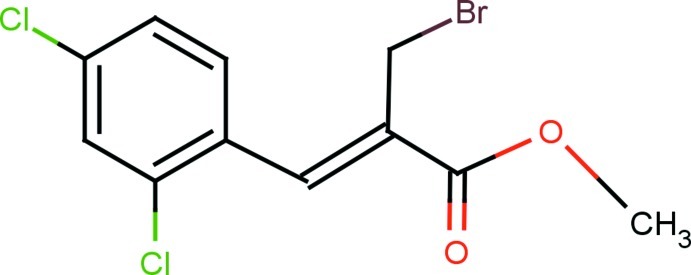


## Experimental

### 

#### Crystal data

C_11_H_9_BrCl_2_O_2_
*M* *_r_* = 323.99
Triclinic, 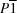

*a* = 7.9174 (3) Å
*b* = 8.8032 (3) Å
*c* = 9.3585 (3) Å
α = 78.374 (2)°
β = 86.599 (2)°
γ = 73.528 (2)°
*V* = 612.67 (4) Å^3^
*Z* = 2
Mo *K*α radiation
μ = 3.77 mm^−1^
*T* = 293 K
0.25 × 0.25 × 0.20 mm

#### Data collection

Bruker Kappa APEXII CCD diffractometer
Absorption correction: multi-scan (*SADABS*; Bruker, 2008 ▶) *T*_min_ = 0.405, *T*_max_ = 0.470
16418 measured reflections
3748 independent reflections
2262 reflections with *I* > 2σ(*I*)
*R*_int_ = 0.033

#### Refinement

*R*[*F* ^2^ > 2σ(*F* ^2^)] = 0.037
*wR*(*F* ^2^) = 0.102
*S* = 1.00
3748 reflections
146 parameters
H-atom parameters constrained
Δρ_max_ = 0.59 e Å^−3^
Δρ_min_ = −0.29 e Å^−3^

### 

Data collection: *APEX2* (Bruker, 2008 ▶); cell refinement: *SAINT* (Bruker, 2008 ▶); data reduction: *SAINT*; program(s) used to solve structure: *SHELXS97* (Sheldrick, 2008 ▶); program(s) used to refine structure: *SHELXL97* (Sheldrick, 2008 ▶); molecular graphics: *ORTEP-3 for Windows* (Farrugia, 2012 ▶) and *Mercury* (Macrae *et al.*, 2008 ▶); software used to prepare material for publication: *SHELXL97* and *PLATON* (Spek, 2009 ▶).

## Supplementary Material

Crystal structure: contains datablock(s) global, I. DOI: 10.1107/S1600536813007368/ld2096sup1.cif

Structure factors: contains datablock(s) I. DOI: 10.1107/S1600536813007368/ld2096Isup2.hkl

Supplementary material file. DOI: 10.1107/S1600536813007368/ld2096Isup3.cml

Additional supplementary materials:  crystallographic information; 3D view; checkCIF report

## Figures and Tables

**Table 1:** Hydrogen-bond geometry (Å, °)

*D*—H⋯*A*	*D*—H	H⋯*A*	*D*⋯*A*	*D*—H⋯*A*
C2—H2⋯O1^i^	0.93	2.33	3.238 (3)	167

Symmetry code: (i) 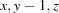.

**Fig. 1.**
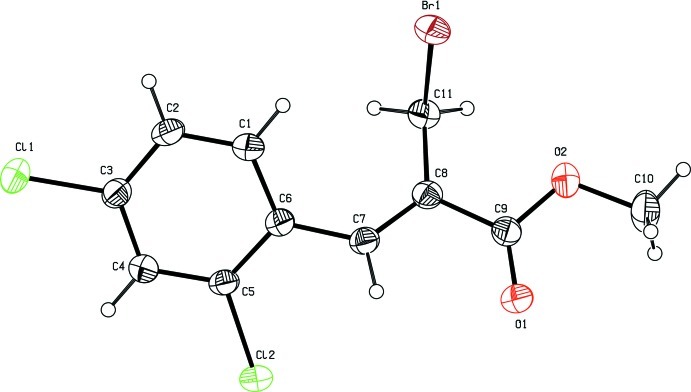
The molecular structure of the title compound with the atom numbering scheme. The displacement ellipsoids are drawn at 30% probability level. H atoms are presented as small spheres of arbitary radius.

**Fig. 2.**
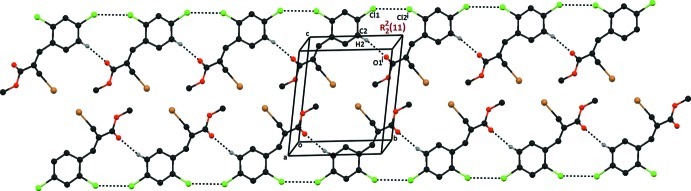
Crystal structure of the title compound, showing the formation of R22(11) ring motifs. Hydrogen bonds are shown as dotted lines. The hydrogen atoms not involved in bonding have been omitted for the sake of clarity.

## supplementary crystallographic information

### Comment

Cinnamic acid and its derivatives are important compounds because of their agrochemical and medical applications (De Fraine & Martin, 1991). Also they possess significant antibacterial activities against *Staphylococcus aureus* (Xiao *et al.* 2008).

X-ray analysis established the molecular structure and atom connectivity of the title compound, as illustrated in Fig. 1. Its methylacrylate part is essentially planar with a maximum deviation of 0.0390 (24) Å for atom C8 and forms dihedral angle of 28.25 (9) ° with the phenyl ring C1–C6. The methylacrylate moiety adopts an extended conformation as evident from the torsion angle values [C7–C8–C9–O1 = 4.7 (4), C7–C8–C9–O2 = 175.2 (2), C8–C9–O2–C10 = -178.8 (2) and O1–C9–O2–C10 = 1.3 (4) °]. The reasons for the extended conformation were discussed earlier (Schweizer and Dunitz, 1982).

The phenyl ring (C1–C6) and the carbonyl group of the acrylate are (+)syn-periplanar to each other with the torsion angle of C7–C8–C9–O1 = 4.7 (4) °. The carbonyl group of the acrylate and the methyl bromide group are (-)anti-periplanar to each other with the torsion angle of C11–C8–C9–O1 = -171.8 (2) °. The least square plane of methyl bromide group forms dihedral angles of 81.44 (17) and 82.48 (15) ° with the phenyl ring and the acrylate group, respectively, being almost orthogonal to both. The chlorine atom Cl1 is slightly deviating from the phenyl ring plane (C1–C6) – by -0.033 (1) Å. The title compound exhibits structural similarities with an earlier reported related structure (Karthikeyan *et al.* 2012).

The crystal packing is stabilized by intermolecular C2—H2···O1^i^ hydrogen bond and halogen interation of type I mode represented by Cl1···Cl2^i^ contact [d = 3.486 (3) Å, θ = 151.68 (6) °] which form *R*_2_^2^(11) ring motifs which in turn, connect the molecules to form bands parallel to [0 1 0] (Schmidt *et al.* 2011, Johnson & Lemmerer, 2012). The symmetry code: (i) *x*,-1 + *y*,*z*. The packing view of the title compound is shown in Fig.2.

### Experimental

To a stirred solution of methyl 2-((2,4-dichlorophenyl)(hydroxy)methyl) acrylate (4 mmol) in CH_2_Cl_2_ (15 ml), 48% aqueous HBr (0.68 ml) was added at room temperature. The reaction mixture was cooled to 273 K and then catalytic amount of concentrated H_2_SO_4_ was added dropwise. The reaction mixture was stirred well at room temperature for about 24 hrs. After the completion of the reaction (confirmed by TLC analysis), the reaction mixture was poured into water and the aqueous layer was extracted with ethyl acetate (3 *x* 10 ml). The combined organic layer was washed with brine (10 ml) and concentrated. The crude product thus obtained was purified by column chromatography (EtOAc/Hexane, 2–6%) to provide (Methyl (2*Z*)-2-(bromomethyl)-3-(2,4-dichlorophenyl) prop-2-enoate) in 93% yield, as a yellow crystalline solid.

### Refinement

Hydrogen atoms were placed in calculated positions with C—H = 0.93 - 0.97 Å and refined in riding model with fixed isotropic displacement parameters: *U*_iso_(H) = 1.2 *U*_eq_(C) for aromatic and methylene groups *U*_iso_(H) = 1.5 *U*_eq_(O) for methyl group. The rotation angles for methyl group were optimized by least squares.

### Crystal data

**Table:**

C_11_H_9_BrCl_2_O_2_	*Z* = 2
*M**_r_* = 323.99	*F*(000) = 320
Triclinic, *P*1	*D*_x_ = 1.756 Mg m^−^^3^
Hall symbol: -P 1	Mo *K*α radiation, λ = 0.71073 Å
*a* = 7.9174 (3) Å	Cell parameters from 3748 reflections
*b* = 8.8032 (3) Å	θ = 2.2–30.6°
*c* = 9.3585 (3) Å	µ = 3.77 mm^−^^1^
α = 78.374 (2)°	*T* = 293 K
β = 86.599 (2)°	Block, yellow
γ = 73.528 (2)°	0.25 × 0.25 × 0.20 mm
*V* = 612.67 (4) Å^3^	

### Data collection

**Table:**

Bruker Kappa APEXII CCD diffractometer	3748 independent reflections
Radiation source: fine-focus sealed tube	2262 reflections with *I* > 2σ(*I*)
Graphite monochromator	*R*_int_ = 0.033
ω and φ scans	θ_max_ = 30.6°, θ_min_ = 2.2°
Absorption correction: multi-scan (*SADABS*; Bruker, 2008)	*h* = −11→11
*T*_min_ = 0.405, *T*_max_ = 0.470	*k* = −12→12
16418 measured reflections	*l* = −13→13

### Refinement

**Table:**

Refinement on *F*^2^	Primary atom site location: structure-invariant direct methods
Least-squares matrix: full	Secondary atom site location: difference Fourier map
*R*[*F*^2^ > 2σ(*F*^2^)] = 0.037	Hydrogen site location: inferred from neighbouring sites
*wR*(*F*^2^) = 0.102	H-atom parameters constrained
*S* = 1.00	*w* = 1/[σ^2^(*F*_o_^2^) + (0.0538*P*)^2^ + 0.0238*P*] where *P* = (*F*_o_^2^ + 2*F*_c_^2^)/3
3748 reflections	(Δ/σ)_max_ = 0.005
146 parameters	Δρ_max_ = 0.59 e Å^−^^3^
0 restraints	Δρ_min_ = −0.29 e Å^−^^3^

### Special details

**Table:**

Geometry. All e.s.d.'s (except the e.s.d. in the dihedral angle between two l.s. planes) are estimated using the full covariance matrix. The cell e.s.d.'s are taken into account individually in the estimation of e.s.d.'s in distances, angles and torsion angles; correlations between e.s.d.'s in cell parameters are only used when they are defined by crystal symmetry. An approximate (isotropic) treatment of cell e.s.d.'s is used for estimating e.s.d.'s involving l.s. planes.
Refinement. Refinement of *F*^2^ against ALL reflections. The weighted *R*-factor *wR* and goodness of fit *S* are based on *F*^2^, conventional *R*-factors *R* are based on *F*, with *F* set to zero for negative *F*^2^. The threshold expression of *F*^2^ > σ(*F*^2^) is used only for calculating *R*-factors(gt) *etc*. and is not relevant to the choice of reflections for refinement. *R*-factors based on *F*^2^ are statistically about twice as large as those based on *F*, and *R*- factors based on ALL data will be even larger.

### Fractional atomic coordinates and isotropic or equivalent isotropic displacement parameters (Å^2^)

**Table:**

	*x*	*y*	*z*	*U*_iso_*/*U*_eq_	
C1	0.2787 (3)	0.5411 (3)	−0.0226 (3)	0.0472 (6)	
H1	0.2484	0.5224	0.0756	0.057*	
C2	0.3057 (3)	0.4177 (3)	−0.0979 (3)	0.0485 (6)	
H2	0.2935	0.3175	−0.0513	0.058*	
C3	0.3509 (3)	0.4437 (3)	−0.2424 (3)	0.0456 (6)	
C4	0.3696 (3)	0.5905 (3)	−0.3132 (3)	0.0449 (5)	
H4	0.4001	0.6071	−0.4115	0.054*	
C5	0.3424 (3)	0.7123 (3)	−0.2360 (2)	0.0409 (5)	
C6	0.2948 (3)	0.6937 (3)	−0.0878 (2)	0.0402 (5)	
C7	0.2786 (3)	0.8231 (3)	−0.0076 (2)	0.0424 (5)	
H7	0.3505	0.8903	−0.0403	0.051*	
C8	0.1750 (3)	0.8592 (3)	0.1064 (2)	0.0416 (5)	
C9	0.1931 (3)	0.9985 (3)	0.1650 (3)	0.0457 (6)	
C10	0.1030 (4)	1.1546 (4)	0.3480 (3)	0.0701 (9)	
H10A	0.2227	1.1438	0.3718	0.105*	
H10B	0.0324	1.1564	0.4349	0.105*	
H10C	0.0585	1.2534	0.2792	0.105*	
C11	0.0395 (3)	0.7785 (3)	0.1709 (3)	0.0468 (6)	
H11A	0.0034	0.7295	0.0983	0.056*	
H11B	−0.0630	0.8590	0.1971	0.056*	
O1	0.2835 (3)	1.0836 (2)	0.1128 (2)	0.0692 (6)	
O2	0.0964 (3)	1.0191 (2)	0.2845 (2)	0.0595 (5)	
Cl1	0.38993 (11)	0.28780 (9)	−0.33786 (9)	0.0670 (2)	
Cl2	0.36663 (10)	0.89643 (7)	−0.32891 (7)	0.05947 (19)	
Br1	0.12451 (4)	0.61222 (3)	0.34428 (3)	0.06038 (13)	

### Atomic displacement parameters (Å^2^)

**Table:**

	*U*^11^	*U*^22^	*U*^33^	*U*^12^	*U*^13^	*U*^23^
C1	0.0551 (14)	0.0448 (14)	0.0415 (13)	−0.0184 (12)	0.0036 (11)	−0.0025 (11)
C2	0.0534 (14)	0.0381 (12)	0.0556 (15)	−0.0199 (11)	0.0025 (11)	−0.0029 (11)
C3	0.0462 (13)	0.0407 (13)	0.0532 (14)	−0.0157 (11)	0.0046 (11)	−0.0124 (11)
C4	0.0493 (13)	0.0464 (14)	0.0414 (12)	−0.0179 (11)	0.0048 (10)	−0.0085 (11)
C5	0.0454 (12)	0.0358 (12)	0.0416 (12)	−0.0158 (10)	0.0019 (10)	−0.0019 (10)
C6	0.0416 (12)	0.0376 (12)	0.0413 (12)	−0.0122 (10)	0.0002 (9)	−0.0059 (10)
C7	0.0494 (13)	0.0381 (12)	0.0404 (12)	−0.0159 (10)	−0.0009 (10)	−0.0036 (10)
C8	0.0465 (12)	0.0391 (12)	0.0363 (12)	−0.0106 (10)	−0.0047 (10)	−0.0009 (10)
C9	0.0576 (14)	0.0415 (13)	0.0359 (12)	−0.0124 (11)	−0.0025 (10)	−0.0038 (10)
C10	0.079 (2)	0.0678 (19)	0.072 (2)	−0.0185 (16)	0.0056 (16)	−0.0375 (17)
C11	0.0469 (13)	0.0488 (14)	0.0442 (13)	−0.0151 (11)	−0.0003 (10)	−0.0054 (11)
O1	0.1119 (17)	0.0591 (12)	0.0510 (11)	−0.0479 (12)	0.0175 (11)	−0.0140 (9)
O2	0.0707 (12)	0.0581 (12)	0.0577 (12)	−0.0226 (10)	0.0141 (9)	−0.0268 (10)
Cl1	0.0846 (5)	0.0527 (4)	0.0762 (5)	−0.0307 (4)	0.0176 (4)	−0.0297 (4)
Cl2	0.0858 (5)	0.0439 (3)	0.0512 (4)	−0.0290 (3)	0.0154 (3)	−0.0039 (3)
Br1	0.0792 (2)	0.0610 (2)	0.04088 (16)	−0.02708 (15)	0.00521 (12)	−0.00037 (12)

### Geometric parameters (Å, º)

**Table:**

C1—C2	1.371 (4)	C7—H7	0.9300
C1—C6	1.397 (3)	C8—C11	1.484 (3)
C1—H1	0.9300	C8—C9	1.485 (3)
C2—C3	1.369 (4)	C9—O1	1.195 (3)
C2—H2	0.9300	C9—O2	1.331 (3)
C3—C4	1.372 (3)	C10—O2	1.451 (3)
C3—Cl1	1.731 (3)	C10—H10A	0.9600
C4—C5	1.372 (3)	C10—H10B	0.9600
C4—H4	0.9300	C10—H10C	0.9600
C5—C6	1.404 (3)	C11—Br1	1.961 (2)
C5—Cl2	1.735 (2)	C11—H11A	0.9700
C6—C7	1.459 (3)	C11—H11B	0.9700
C7—C8	1.340 (3)		
			
C2—C1—C6	122.5 (2)	C6—C7—H7	115.3
C2—C1—H1	118.7	C7—C8—C11	125.5 (2)
C6—C1—H1	118.7	C7—C8—C9	115.8 (2)
C3—C2—C1	119.2 (2)	C11—C8—C9	118.6 (2)
C3—C2—H2	120.4	O1—C9—O2	122.9 (2)
C1—C2—H2	120.4	O1—C9—C8	124.8 (2)
C2—C3—C4	121.3 (2)	O2—C9—C8	112.3 (2)
C2—C3—Cl1	119.7 (2)	O2—C10—H10A	109.5
C4—C3—Cl1	118.97 (19)	O2—C10—H10B	109.5
C5—C4—C3	118.6 (2)	H10A—C10—H10B	109.5
C5—C4—H4	120.7	O2—C10—H10C	109.5
C3—C4—H4	120.7	H10A—C10—H10C	109.5
C4—C5—C6	122.9 (2)	H10B—C10—H10C	109.5
C4—C5—Cl2	117.39 (18)	C8—C11—Br1	112.60 (15)
C6—C5—Cl2	119.75 (18)	C8—C11—H11A	109.1
C1—C6—C5	115.5 (2)	Br1—C11—H11A	109.1
C1—C6—C7	123.4 (2)	C8—C11—H11B	109.1
C5—C6—C7	120.9 (2)	Br1—C11—H11B	109.1
C8—C7—C6	129.4 (2)	H11A—C11—H11B	107.8
C8—C7—H7	115.3	C9—O2—C10	116.2 (2)
			
C6—C1—C2—C3	0.2 (4)	C1—C6—C7—C8	−34.5 (4)
C1—C2—C3—C4	0.0 (4)	C5—C6—C7—C8	150.7 (2)
C1—C2—C3—Cl1	178.65 (19)	C6—C7—C8—C11	−5.0 (4)
C2—C3—C4—C5	0.1 (4)	C6—C7—C8—C9	178.8 (2)
Cl1—C3—C4—C5	−178.53 (18)	C7—C8—C9—O1	4.7 (4)
C3—C4—C5—C6	−0.5 (4)	C11—C8—C9—O1	−171.8 (2)
C3—C4—C5—Cl2	−179.85 (18)	C7—C8—C9—O2	−175.2 (2)
C2—C1—C6—C5	−0.5 (4)	C11—C8—C9—O2	8.4 (3)
C2—C1—C6—C7	−175.6 (2)	C7—C8—C11—Br1	98.2 (2)
C4—C5—C6—C1	0.7 (3)	C9—C8—C11—Br1	−85.7 (2)
Cl2—C5—C6—C1	−179.97 (18)	O1—C9—O2—C10	1.3 (4)
C4—C5—C6—C7	175.9 (2)	C8—C9—O2—C10	−178.8 (2)
Cl2—C5—C6—C7	−4.7 (3)		

### Hydrogen-bond geometry (Å, º)

**Table:**

*D*—H···*A*	*D*—H	H···*A*	*D*···*A*	*D*—H···*A*
C2—H2···O1^i^	0.93	2.33	3.238 (3)	167

Symmetry code: (i) *x*, *y*−1, *z*.
